# Estimated SARS-CoV-2 Seroprevalence Among Persons Aged <18 Years — Mississippi, May–September 2020

**DOI:** 10.15585/mmwr.mm7009a4

**Published:** 2021-03-05

**Authors:** Charlotte V. Hobbs, Jan Drobeniuc, Theresa Kittle, John Williams, Paul Byers, Panayampalli S. Satheshkumar, Kengo Inagaki, Meagan Stephenson, Sara S. Kim, Manish M. Patel, Brendan Flannery, CDC COVID-19 Response Team, CDC COVID-19 Response Team, Bailey Alston, Shanna J. Bolcen, Darbi Boulay, Peter Browning, Li Cronin, Ebenezer David, Tonya Hayden, Han Li, Travis Lim, Panagiotis Maniatis, Palak Patel, Mathew Pauly, Amanda Poe, Lili Punkova, Vera Semenova, Evelene P. Steward-Clark, Alexandra Tejada, Briana Zellner

**Affiliations:** ^1^Children’s of Mississippi, University of Mississippi Medical Center, Jackson, Mississippi; ^2^CDC COVID-19 Response Team; ^3^Mississippi State Department of Health.; CDC; CDC; CDC; CDC; CDC; CDC; CDC; CDC; CDC; CDC; CDC; CDC; CDC; CDC; CDC; CDC; CDC; CDC.

As of March 1, 2021, persons aged <18 years accounted for approximately 11% of 28.4 million reported COVID-19 cases in the United States*; however, data on pediatric infections with SARS-CoV-2, the virus that causes COVID-19, are limited (*1*). Surveys of SARS-CoV-2 antibody seroprevalence suggest that cumulative incidence of infection is much higher than that ascertained by reported COVID-19 cases (*2*,*3*). Evidence of previous SARS-CoV-2 infections among young persons in Mississippi was assessed by testing for antibodies to SARS-CoV-2 on a convenience sample of residual serum specimens collected for routine testing by an academic medical center laboratory during May 17–September 19, 2020. Seroprevalence by calendar month was standardized to the state population by race/ethnicity; cumulative numbers of infections were estimated by extrapolating seroprevalence to all persons aged <18 years in Mississippi. Serum specimens from 1,603 persons were tested; 175 (10.9%) were positive for SARS-CoV-2 antibodies. Among 1,579 (98.5%) specimens for which the race/ethnicity of the person tested was known, specimens from 16 (23.2%) of 69 Hispanic persons, 117 (13.0%) of 901 non-Hispanic Black persons, and 30 (5.3%) of 565 non-Hispanic White persons tested positive. Population-weighted seroprevalence estimates among persons aged <18 years increased from 2.5% in May to 16.3% in September 2020. Based on these estimates, 113,842 (95% confidence interval [CI] = 90,096–153,652) persons aged <18 years in Mississippi might have been infected with SARS-CoV-2 by mid-September 2020. The number of COVID-19 cases reported in this age group through August 31, 2020 was 8,993. Serosurveys that include pediatric age groups can help provide evidence of cumulative disease incidence, estimate frequency of undiagnosed cases of SARS-CoV-2 among young persons, and guide prevention efforts.

Most persons who are infected with SARS-CoV-2 develop antibodies to SARS-CoV-2 proteins within 1–2 weeks of disease onset (*4*). Serologic testing for SARS-CoV-2 antibodies, albeit having imperfect sensitivity and specificity,^†^ is useful to identify past SARS-CoV-2 infections. Serology tests are used widely in seroprevalence studies to understand patterns of virus spread and cumulative incidence of SARS-CoV-2 infection^§^ (*2*,*3*).

This retrospective seroprevalence study was conducted by the University of Mississippi Medical Center in collaboration with the Mississippi State Department of Health (MSDH) and CDC to describe trends in SARS-CoV-2 antibody seroprevalence among young persons in Mississippi during the COVID-19 pandemic. The University of Mississippi Medical Center provides clinical laboratory services for university hospitals in central Mississippi and 12 hospitals outside the university network statewide (*5*). Demographic data including age, sex, race/ethnicity, and date of collection were obtained for deidentified residual serum specimens collected for routine clinical testing during May 17–September 19, 2020, from persons aged <18 years. One specimen per person was included in the analysis, either the first seropositive specimen or the earliest specimen from persons with all seronegative specimens, to avoid potential bias in underestimating infections from decline in antibodies below the limit of detection for seropositivity. Sera were stored at −20°C (−4°F) before testing at CDC.

Seropositivity was determined for serum specimens using one of two assays, based on specimen volume. Specimens with adequate volume (≥0.3 mL) were tested with a qualitative VITROS anti–SARS-CoV-2 total antibody in vitro diagnostic test using the automated VITROS 3600 Immunodiagnostic System (Ortho Clinical Diagnostics) (*6*). One aliquot was heat-treated at 56°C (132.8°F) for 10 minutes and tested on the VITROS Immunodiagnostic System. An automatically calculated ratio of test sample signal to cutoff value (S/C) <1.0 was interpreted as nonreactive, and S/C ≥1.0 was interpreted as reactive for anti–SARS-CoV-2 total antibody (*6*). Samples with volumes <0.3 mL were tested to determine seropositivity using an enzyme linked immunosorbent assay (ELISA) developed by CDC to measure total SARS-CoV-2 antibodies against the extracellular domain of the SARS-CoV-2 spike protein (*2*).^¶^

Seroprevalence by calendar month was standardized to the Mississippi population aged <18 years by race/ethnicity**; 95% CIs accounting for assay test performance were estimated by using published methods (*2*). Cumulative numbers of SARS-CoV-2 infections were estimated by extrapolating seroprevalence and 95% CIs to the Mississippi population aged <18 years and were compared with cumulative numbers of confirmed and probable COVID-19 cases (as defined by the Council of State and Territorial Epidemiologists)^††^ in persons aged <18 years reported to MSDH.^§§^ Ratios of estimated SARS-CoV-2 infections to reported COVID-19 cases were calculated by dividing estimated numbers of SARS-CoV-2 infections by the reported cumulative number of COVID-19 cases as of the last day of the preceding month. Statistical analyses were conducted using SAS (version 9.4; SAS Institute). This activity was reviewed by CDC and was conducted consistent with applicable federal law and CDC policy.^¶¶^ The project was also reviewed and approved by the University of Mississippi Medical Center Institutional Review Board through its expedited review procedure.

Among 1,603 serum specimens from persons aged <18 years included in analyses, 175 (10.9%) tested positive for SARS-CoV-2 antibodies, including 152 of 1,469 (10.4%) by VITROS assay and 23 of 134 (17.2%) by ELISA (Table 1). Among 1,579 (98.5%) specimens for which the race/ethnicity of the person receiving testing was known, specimens from 16 (23.2%) of 69 Hispanic persons, 117 (13.0%) of 901 non-Hispanic Black persons, and 30 (5.3%) of 565 non-Hispanic White persons tested seropositive. After adjusting by race/ethnicity to the Mississippi population aged <18 years, estimated seroprevalence increased from 2.5% in May to 16.3% in September (Figure). Extrapolating to the state population, an estimated 113,842 (95% CI = 90,096–153,652) Mississippi residents aged <18 years might have been infected with SARS-CoV-2 by mid-September 2020; through August 31, a total of 8,993 COVID-19 cases among persons aged <18 years had been reported to MSDH (Table 2). Ratios of estimated numbers of SARS-CoV-2 cases based on seropositivity to COVID-19 cases reported at the end of the preceding month decreased from 68.2 in May to 12.7 in September. This finding suggests an improvement in case detection over time, even though the number of SARS-CoV-2 cases estimated from seroprevalence was consistently higher than the number of SARS-CoV-2 cases reported during each month.

**TABLE 1:** Characteristics and SARS-CoV-2 serology results of persons aged <18 years whose residual serum specimens* were tested for presence of SARS-CoV-2 antibodies — Mississippi, May 17–September 19, 2020

Characteristic	Total	SARS-CoV-2 serology result		P-value^§^
		No. positive	% (95% CI)^†^	
**Overall**	**1,603**	175	10.9 (9.4–12.4)	—
**Age group**				0.03
<6 mos	**420**	61	14.5 (11.2–17.9)	
6–11 mos	**63**	9	14.3 (5.6–22.9)	
1–8 yrs	**423**	42	9.9 (7.1–12.8)	
9–17 yrs	**697**	63	9.0 (6.9–11.2)	
**Sex (missing = 2)**				0.28
Female	**771**	91	11.8 (9.6–14.1)	
Male	**830**	84	10.1 (8.1–12.2)	
**Race/Ethnicity (missing = 24)**				<0.01
Black, non-Hispanic	**901**	117	13.0 (10.8–15.2)	
Hispanic	**69**	16	23.2 (13.2–33.2)	
Other, non-Hispanic	**44**	7	15.9 (5.1–26.7)	
White, non-Hispanic	**565**	30	5.3 (3.5–7.2)	
**Assay**				0.02^¶^
Ortho VITROS	**1,469**	152	10.4 (8.8–11.9)	
CDC ELISA	**134**	23	17.2 (10.8–23.6)	
**Dates of specimen collection**				<0.01
May 17–May 31	**174**	6	3.5 (0.7–6.2)	
Jun 1–30	**447**	28	6.3 (4.0–8.5)	
Jul 1–31	**339**	35	10.3 (7.1–13.6)	
Aug 1–31	**368**	56	15.2 (11.6–18.9)	
Sep 1–19	**275**	50	18.2 (13.6–22.7)	

**Abbreviations:** CI = confidence interval; ELISA = enzyme-linked immunosorbent assay.

* Includes residual serum specimens from 1,603 persons aged <18 years.

^†^ 95% CIs for seroprevalence by month were calculated assuming a binomial distribution for SARS-CoV-2 seropositivity in the sample.

^§^ P-value for difference in proportion seropositive by individual characteristic, serologic assay, or dates of specimen collection using chi-squared text.

^¶^ P-value for difference in proportion seropositive in specimens tested by each assay; individual specimens were tested with Ortho VITROS if specimen volume was ≥0.3 mL or CDC ELISA if specimen volume was <0.3 mL.

**FIGURE:**
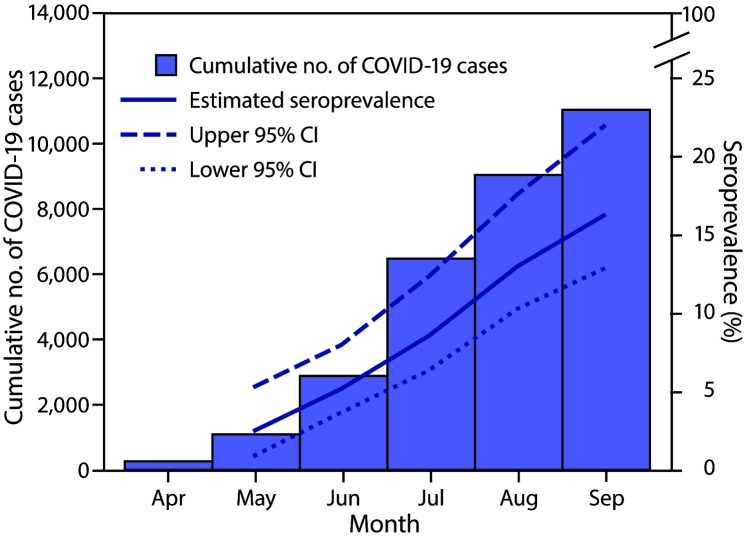
Cumulative number of reported COVID-19 cases and estimated race/ethnicity–standardized SARS-CoV-2 antibody seroprevalence* among persons aged <18 years — Mississippi, April–September 2020 **Abbreviation:** CI = confidence interval. * From residual serum specimens collected during May 17–September 19, 2020, from persons aged <18 years.

**TABLE 2:** Estimated number of SARS-CoV-2 infections based on seroprevalence estimates and the number of reported COVID-19 cases at of the end of the preceding month among persons aged <18 years — Mississippi, May 17–September 19, 2020

Specimen collection date	Estimated seroprevalence* % (95% CI)	Estimated cumulative infections,^†^ no. (95% CI)	Cumulative reported cases^§^ (report date)	Estimated infection/reported case ratio (95% CI)
May 17–31	2.5 (0.9–5.3)	17,461 (6,286–37,016)	256 (Apr 30)	68.2 (24.6–144.6)
Jun 1–30	5.2 (3.7–8.0)	36,318 (25,842–55,874)	1,083 (May 31)	33.5 (23.9–51.6)
Jul 1–31	8.6 (6.4–12.4)	60,064 (44,699–86,604)	2,869 (Jun 30)	20.9 (15.6–30.2)
Aug 1–31	13.0 (10.3–17.6)	90,795 (71,937–122,922)	6,439 (Jul 31)	14.1 (11.2–19.1)
Sep 1–19	16.3 (12.9–22.0)	113,842 (90,096–153,652)	8,993 (Aug 31)	12.7 (10.0–17.1)

**Abbreviation:** CI = confidence interval.

* Standardized to the race/ethnicity distribution of the Mississippi population aged <18 years using weights derived from the 2019 U.S. Census; 95% CIs were calculated for race/ethnicity–weighted SARS-CoV-2 seropositivity in the sample accounting for assay test performance.

**^†^** Race/ethnicity–standardized seroprevalence estimates for each period applied to the 2019 Mississippi population aged <18 years = 698,420 (https://www.census.gov).

**^§^** Cumulative number of confirmed and probable COVID-19 cases in persons aged <18 years reported to Mississippi State Department of Health as of the end of the preceding month.

## Discussion

During July–August 2020, Mississippi experienced a rapid rise in COVID-19 cases, which preceded a second peak in incidence in December 2020. Analyses of a convenience sample of residual sera collected during May–September 2020, indicated that 16.3% of children and adolescents in Mississippi might have been infected with SARS-CoV-2 by mid-September 2020. Seropositivity rates among non-Hispanic Black and Hispanic young persons were 2.4 and 4.3 times, respectively, the rate among non-Hispanic White persons. Monthly increases in population-weighted seroprevalence among persons aged <18 years paralleled increases in the cumulative number of reported COVID-19 cases among young persons in Mississippi. Projected cumulative infections based on seroprevalence suggests that case-based surveillance underestimated SARS-CoV-2 infections among children and adolescents, consistent with national data suggesting underascertainment of COVID-19 disease incidence in all age groups***^,^^†††^ (*7*). Compared with seroprevalence data from older age groups in Mississippi, data from this study sample suggests that cumulative infection rates by mid-September among persons aged <18 years were similar to those among persons aged 18–49 years, the age group with the highest seroprevalence during the period (*3*).

Nationwide serosurveys have identified varying seroprevalences by sex, age group, and urban/rural status. In four cross-sectional serosurveys conducted in Mississippi during July–October 2020, female sex, age 18–49 years, and living in nonmetropolitan jurisdictions were associated with higher SARS-CoV-2 seroprevalence (*3*). However, numbers of specimens from persons aged <18 years were insufficient to provide seroprevalence estimates for this age group. In contrast, the current investigation of percentage of serum specimens with positive SARS-CoV-2 antibody test results benefited from large numbers of pediatric specimens collected during a 4-month period when incidence of reported COVID-19 cases increased rapidly.

The findings in this report are subject to at least four limitations. First, seroprevalence among a convenience sample of sera from one laboratory might not be representative of seroprevalence among all persons aged <18 years in Mississippi; therefore, actual numbers of SARS-CoV-2 infections in this age group might have been higher or lower than projected. Second, young persons who have blood collected for routine laboratory testing might differ from the general pediatric population with respect to underlying health conditions, access to care, or adherence to prevention measures including use of masks and physical distancing. However, compared with more representative serosurveys, residual sera from commercial laboratories have previously been shown to provide an approximate measure of community SARS-CoV-2 seroprevalence (*3*). Third, misclassification of antibody status was possible because of imperfect sensitivity and specificity of the assays used in the report. Finally, selecting the first seropositive specimen from persons receiving positive test results at any time point rather than randomly selected specimens might have overestimated population seroprevalence. Alternatively, seroprevalence could be underestimated if participants who were infected had not yet mounted an antibody response or if antibody titers had declined since infection (*8*,*9*).

These estimates of SARS-CoV-2 infections among children and adolescents in Mississippi add to those from other studies of the general population from nationwide cross-sectional serosurveys. Including pediatric age groups in serosurveys can help track the spread of SARS-CoV-2 among young persons in the United States.

**Summary**

**What is already known about the topic?**

Serosurveys estimating prior SARS-CoV-2 infections in the United States have focused on adults; little is known about seroprevalence among young persons.

**What is added by this report?**

Serologic testing of residual blood specimens collected during May–September 2020, from 1,603 persons aged <18 years suggested that approximately 113,842 (16.3%) of 698,420 young persons in Mississippi might have been infected with SARS-CoV-2 by mid-September 2020, and only 8,993 confirmed and probable COVID-19 cases among young persons had been reported to the Mississippi State Department of Health by August 31.

**What are the implications for public health practice?**

Serosurveys including pediatric age groups help estimate cumulative disease incidence and frequency of undiagnosed cases of COVID-19 among young persons to guide prevention efforts.

## Footnotes

*
https://covid.cdc.gov/covid-data-tracker/#demographics

†
https://www.cdc.gov/coronavirus/2019-ncov/lab/resources/antibody-tests-guidelines.html

§
https://covid.cdc.gov/covid-data-tracker/#national-lab

¶
https://www.biorxiv.org/content/10.1101/2020.04.24.057323v2

** 2019 U.S. Census data for Mississippi. https://wonder.cdc.gov

††
https://wwwn.cdc.gov/nndss/conditions/coronavirus-disease-2019-covid-19/case-definition/2020/

§§
https://msdh.ms.gov/msdhsite/_static/14,0,420.html

¶¶ Activity was determined to meet the requirements of public health surveillance as defined in 45 CFR 46.102(l)(2).

***
https://www.cdc.gov/coronavirus/2019-ncov/cases-updates/burden.html

†††

https://www.medrxiv.org/content/10.1101/2021.01.27.21250570v1.full
